# Vitamin D Deficiency, Osteoporosis and Effect on Autoimmune Diseases and Hematopoiesis: A Review

**DOI:** 10.3390/ijms22168855

**Published:** 2021-08-17

**Authors:** Massimo De Martinis, Alessandro Allegra, Maria Maddalena Sirufo, Alessandro Tonacci, Giovanni Pioggia, Martina Raggiunti, Lia Ginaldi, Sebastiano Gangemi

**Affiliations:** 1 Department of Life, Health and Environmental Sciences, University of L’Aquila, 67100 L’Aquila, Italy; demartinis@cc.univaq.it (M.D.M.); maddalena.sirufo@gmail.com (M.M.S.); martinaraggiunti@libero.it (M.R.); lia.ginaldi@univaq.it (L.G.); 2 Allergy and Clinical Immunology Unit, Center for the Diagnosis and Treatment of Osteoporosis, AUSL 04 Teramo, 64100 Teramo, Italy; 3 Department of Human Pathology in Adulthood and Childhood “Gaetano Barresi”, Division of Haematology, University of Messina, 98125 Messina, Italy; 4 Clinical Physiology Institute, National Research Council of Italy (IFC-CNR), 56124 Pisa, Italy; atonacci@ifc.cnr.it; 5 Institute for Biomedical Research and Innovation (IRIB), National Research Council of Italy (CNR), 98164 Messina, Italy; giovanni.pioggia@cnr.it; 6 Department of Clinical and Experimental Medicine, School and Operative Unit of Allergy and Clinical Immunology, University of Messina, 98125 Messina, Italy; gangemis@unime.it

**Keywords:** vitamin D, osteoporosis, autoimmune diseases, allergy, endocrinological diseases, monoclonal gammopathies, bone marrow transplantation

## Abstract

Vitamin D (VD) is essential for bone homeostasis, but it is also involved in pleiotropic effects on various organs and tissues. In adults, VD deficiency can cause or exacerbate osteoporosis and induce osteomalacia. However, every tissue and cell in the body has a VD receptor, including the brain, heart, stomach, pancreas, skin, gonads, and immune cells, and a deficiency may modify the function of these organs. Thus, the wide-ranging actions of VD help to explain why a reduction in VD amount has been correlated with numerous chronic diseases. In fact, VD deficiency increases the risk of osteoporosis and several other diseases and complications characterized by impaired bone metabolisms, such as autoimmune diseases, inflammatory bowel diseases, allergy, endocrinological diseases, hematological malignancies, and bone marrow transplantation. This review aims to investigate the link between VD deficiency, osteoporosis, and its concomitant diseases. Further epidemiological and mechanistic studies are necessary in order to ascertain the real role of hypovitaminosis in causing the reported diseases; however, adequate vitamin supplementation and restoration of metabolic normality could be useful for better management of these pathologies.

## 1. General Considerations on Vitamin D

VD is a fat-soluble substance of steroidal nature, its sources can be endogenous, and its production is UVB dependent or exogenous through nutritional intake and supplements [1,2]. Cholecalciferol, the inactive form of VD, requires two hydroxylations for activation: the first takes place in the liver where it is converted into calcidiol or 25-hydroxycholecalciferol (25(OH)D), and the second in the kidney, under strict control of PTH and the phosphaturic hormone fibroblast growth factor 23 (FGF-23), where calcidiol is metabolized into calcitriol or 1-α,25-dihydroxycholecalciferol (1,25(OH)2D) [3]. Serum 25(OH)D levels are positively influenced by ultraviolet radiation in sunlight, while in the liver, kidney, and/or intestine diseases, several drugs and obesity negatively regulate VD levels [4,5,6]. 25(OH)D deficiency can have health repercussions, so it is essential to determine optimal serum calcidiol levels, whereas levels <20 ng/mL have been associated with a greater risk of various disorders [7,8,9].

Although the available scientific data support the key roles of calcium and VD in skeletal health, the evidence has not yet established that nutrients provide benefits or improve extraskeletal health outcomes. A public health report on the dietary intake of calcium and VD by the Institute of Medicine (IOM), released on 30 November 2010, showed that existing data suggest that almost all individuals’ intake levels (recommended dietary dose) of 25(OH)D are at least 20 ng/mL (50 nmol/L), even under conditions of minimal sun exposure [10]. Moreover, there is not yet a standardized laboratory measurement of 25(OH)D because of the variations in analytical techniques and assays (specifically 15–20% variability between different methods). This might have significant implications for research into VD and its role in health and disease, which relies on the accurate assessment of VD status [11,12,13]. The committee also found that the prevalence of VD inadequacy in the North American population has been overestimated by some groups due to the use of inappropriate cut-points, and additional research is needed in order to achieve a consensus regarding clinical and public health cut-points for serum 25(OH)D inadequacy to avoid problems of undertreatment or overtreatment [11,13,14,15].

### 1.1. Vitamin D and Osteoporosis

It is well known that VD promotes calcium absorption in the gut, and it is important to maintain adequate serum calcium concentrations, indispensable for the normal mineralization of the bone [16]. VD is involved in bone growth and bone remodeling by osteoblasts and osteoclasts [17], and its deficiency (25(OH)D < 50 nmol/L) accelerates bone turnover, bone loss, and osteoporotic fractures [18]. In a meta-analysis of 12 double-blinded trials including individuals aged 65 years or older, the antifracture efficacy of supplemental VD significantly increased with a high received dose of VD for nonvertebral fractures and hip fractures. In this study, no fracture reduction was observed for a received dose of 400 IU/d or less; indeed, a high received dose of 482–770 IU/d of supplemental VD reduced nonvertebral fractures by 20% and hip fractures by 18% [16]. Moreover, in a meta-analysis, Tang et al. suggested that a daily intake of at least 800 IU of VD associated with calcium supplementation increases total fracture reduction by 3% compared with daily doses of VD of less than 800 IU [19].

Combined calcium and VD supplementation is significantly related to reduced total fractures and hip fractures across various populations. Although the decrease in risks for hip fractures was stronger than that for total fractures, constantly reduced risks were observed for both fracture outcomes [20]. To date, intermittent or daily administration of standard doses of VD alone has not been associated with a reduced risk or prevention of fracture among adults aged 50 years and older without VD deficiency and OP [21,22,23,24,25,26,27]. Further studies demonstrated that treatment with VD for 3 years at a dose of 4000 IU per day or 10,000 IU per day, compared with 400 IU per day, did not support the benefit of high-dose VD supplementation for bone health [28] and physical performance of lower extremity [29].

Several studies demonstrate that the safety profile of VD supplementation is similar for doses of 400, 4000, and 10,000 IU/day. Hypercalciuria was common and occurred more frequently with higher doses, while hypercalcemia was rare, mild, and transient [30]. Supplementation with ≥2800 IU/d for one year or longer did not significantly increase the risk of total adverse events or of kidney stones but demonstrated a borderline increased risk of hypercalcemia from long-term, high-dose VD supplementation, which requires further investigation [31]. A safety upper limit of 4000 IU/day, which is consistently accepted, has been challenged, since the risk of adverse events in other systems than calcium–phosphate homeostasis may depend not only on the dose but also on the outcome; the treatment regimen; and possibly the patient’s age, sex, and VD status [32]. Nevertheless, it is difficult to draw conclusions regarding kidney stones and hypercalciuria due to the limited number of these events in the viewed studies [31].

### 1.2. Vitamin D and Systemic Diseases

About 200 million people worldwide suffer from osteoporosis (OP), a bone disease linked to an altered bone remodeling that leads to an increase in bone resorption with a consequent increase in fractures. The risk of OP increases with age, and it is greater in postmenopausal women due to estrogen deficiency. In 27 European countries, the prevalence of osteoporosis is 5.5% (22.1% in women and 6.6% in men). Diagnosis of OP is made using dual-energy X-ray absorptiometry (DEXA), which values the bone mineral density (BMD) in two sites, typically the lumbar spine and proximal femur. The pathogenetic mechanism of OP involves several factors, including defects in the trabecular rearrangement, abnormal bone tissue, excessive remodeling, and bone repair deficiency [33]. Non-modifiable factors in the pathogenesis of OP include genetic predisposition and family history, and the modifiable factors are low body mass index (BMI), smoking habit, estrogen deficiency, inadequate calcium intake from diet, physical activity, alcoholism, comorbidities, and low VD levels [34].

The pathogenesis of OP is linked to an altered compensation in favor of bone resorption mediated by osteoclasts compared to a physiological condition in which there is a balance in bone metabolism [35,36]. Activation of osteoclasts requires complex signaling mechanisms between cells of the osteoclast line, mesenchymal cells, and lymphocytes [37,38]. Interactions are controlled by factors including NF-kB ligand (RANKL). The binding of RANKL to the RANK receptor, expressed on the precursors of osteoclasts, determines their differentiation into osteoclasts [39]. In order to normalize the serum calcium levels in conditions of hypocalcemia, VD induces a strong expression of RANKL that results in an increase in bone resorption. In a condition of 25(OH)D deficiency, the low calcium concentration induces an increase in parathormone (PTH), which, through considerable renal reabsorption, increase in 1,25(OH)2D production, and interaction with RANKL, restores the serum calcium values. This mobilization of calcium from the bones reduces their mineral density, increasing the likelihood of OP.

Although 25(OH)D deficiency represents one of the central moments in the onset of OP, this condition appears to be relevant for the pathogenesis of numerous pathological conditions characterized by impaired bone metabolism, osteoporosis, or bone lesions. In fact, 25(OH)D is involved in a wide range of physiological functions, and its deficit has often been associated with several pathologies, including autoimmune, endocrinological, and cardiovascular diseases; some tumors; infections; and alteration to calcium metabolism. 25(OH)D is able to regulate the inflammation response promoting the cyclin-dependent kinase (CDK) inhibitor synthesis, influencing several growth factors and their signaling pathways, such as insulin-like growth factor 1 (IGF-1), transforming growth factor β (TGFβ), Wnt/β-catenin, MAP kinase 5 (MAPK5), and nuclear factor κB (NF-κB); promoting pro-apoptotic mechanisms; inducing differentiation; and regulating androgen and estrogen receptor signaling [40,41]. These activities lead to the containment of autoimmune inflammation and induce differentiation of immune cells in a way that promotes self-tolerance [42]. Many epidemiological studies suggest the link between 25(OH)D deficiency and a higher incidence of autoimmune diseases (Ads) and the role of VD supplementation in the prevention and progression of these pathologies [43,44,45,46]. 25(OH)D is also involved in the maintenance of adequate concentrations of minerals for metabolic functions and bone mineralization, regulating calcium and phosphorus metabolism. Therefore, the role of 25(OH)D is central to the development of OP secondary to rheumatic pathologies, endocrine–metabolic diseases, nutritional deficiencies, renal dysfunctions, and hematological disorders. Moreover, there are several hematological illnesses that might have a detrimental effect on bone structure. Several reports noted bone alterations in subjects affected by neoplastic and non-neoplastic hematological diseases, such as systemic mastocytosis, monoclonal gammopathies, sickle cell disease, hemophilia, and thalassemia, and in patients undergoing bone marrow transplantation (BMT) [47].

In hematological illness, several factors could intervene in the pathogenesis, and anemia may also be correlated with bone frailty [48]. Among the possible reasons for this relationship is the notion that hypoxia may be essentially responsible, as it is a powerful controller of erythropoietin generation that activates osteoclast differentiation and causes bone loss [49]. Iron scarcity may also alter the bone structure, as it is an indispensable component of the hydroxylation of prolyl and lysil residues of procollagen and contributes to VD metabolism via the cytochrome P450 [50]. Numerous hematological neoplasms seem to be characterized by VD deficiency, which could be involved in both bone alterations and in the onset and progression of neoplasms. 25(OH)D deficiency also causes a mineralization defect that can lead to spontaneous fractures and is also a risk factor for diabetes, rheumatoid arthritis, cancer, psychiatric conditions, and several other diseases. Therefore, the assessment of 25(OH)D deficiency and its periodic measurement is currently deemed good clinical practice.

This review aims to investigate the link between 25(OH)D deficiency, comorbidities, and osteoporosis.

## 2. Autoimmune Diseases, VD Deficiency and OP

**Figure 1:**
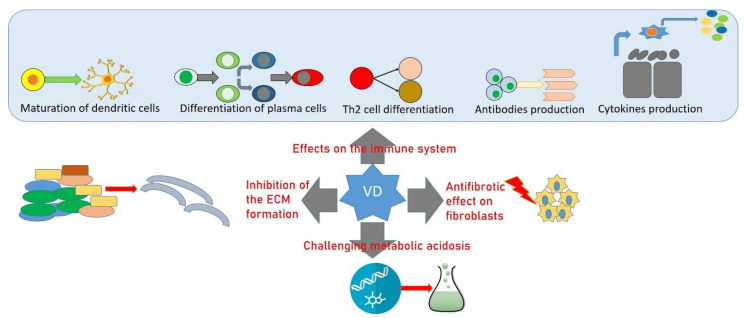
Vitamin D, bone, and autoimmune diseases. ECM: extra cellular matrix; Th2: T helper 2 cells.

The evidence of the immunomodulatory effect of 25(OH)D and the role of VD deficiency and supplementation in Ads has long been studied [51,52,53,54]. Some studies correlated VD dietary intake and the prevalence of Ads [55,56,57] with a correct evaluation of VD intake based on patient response. In addition, 25(OH)D has also been implicated in the control of other inflammatory conditions, as VD supplementation reduces macrophage production of pro-inflammatory cytokines [58,59] and the development of some Ads [60]. Calcitriol can modulate the activity of all cells of the immune system that express the VD receptor (VDR) [61], and it has an immunoregulatory and tolerogenic role because it can influence the maturation and migration of different dendritic cell (DC) subtypes and their production of cytokines and chemokines by binding VDR (Figure 1). This link determines the arrest of the differentiation and maturation of DCs and, therefore, the production of pro-inflammatory cytokines (IL-6, IL-12, and IL-23) and of the tumor necrosis factor-α (TNF-α); it increases the production of anti-inflammatory cytokines (IL-8 and IL-10); it decreases the expression of the main histocompatibility complexes of class I and II and of surface costimulatory molecules (CD40, CD80, CD83, and CD86) [62]; and it inhibits the differentiation of B cells in plasma cells and their antibody production. VD can also act on activated T lymphocytes that express VDR by reducing the activity of Th1 and Th17, promoting the differentiation of Th2 into CD4 + producing IL-4. Calcitriol also promotes the activity of regulatory cells by suppressing the immune response. These effects on immune cells may explain the beneficial effect of VD supplementation observed in Ads [63]. Correlation between 25(OH)D deficiency and Ads is particularly evident in rheumatoid arthritis (RA), systemic lupus erythematosus (SLE), psoriatic arthritis (PsA), systemic sclerosis (SSc), and inflammatory bowel disease (IBD), and it is responsible for an increased risk of secondary OP.

RA 25(OH)D deficiency is inversely associated with pathological activity because the inhibition of monocyte differentiation into DC induced by 25(OH)D reduces the number of antigen-presenting cells available to stimulate T cells, which play a key role in RA [64]. In addition, 1,25(OH)2D inhibits B cell proliferation before differentiation to immunoglobulin-secreting cells, reduces polyclonal and anti-dsDNA IgG immunoglobulin production [65], and contributes to immune tolerance by affecting both the innate and adaptative immune responses [66]. The supplementation of VD at higher doses (but non-toxic) could also be useful to improving the pathological activity of RA.

SLE data regarding VD supplementation are inconsistent [67]. The persistent inflammation associated with physical inactivity, sunshine avoidance, renal impairment, and low levels of 25(OH)D is also involved in the pathogenesis of OP secondary to SLE. These patients are more prone to fractures despite normal bone density [68,69]. In particular, BMD reduction, osteoporosis, and fragility fractures are prevalent in patients with SLE, especially in the lumbar spine when compared to the femur and hip [70,71,72,73].

PsA involves joints and entheses, with immune-mediated pathogeneses [74] affecting 20–30% of patients with psoriasis. The prevalence between men and women is the same with onset among those 40–50 years old [75]. Patients with PsA and psoriasis have more risk factors predisposing to OP and fractures, including diabetes, alcohol abuse, drug use (antidepressants, corticosteroids, methotrexate, and cyclosporine), smoking, depression [76], and an increase in the incidence of fractures of 7–26% compared to the general population. The role of VD in PsA is controversial; in fact, only some studies provide evidence of a correlation between VD deficiency and PsA severity [77], despite the role of VD in decreasing the production of IL-2, IL-6, and interferon-gamma and reducing the inflammatory pathway promoting suppressor T-cell activity being widely demonstrated. However, subjects affected by PsA show low serum 25(OH)D levels, with concentrations lower than 20 ng/mL, and, in some studies, OP is one of the major comorbidities of PsA, with a prevalence of 1.4–68.8% [78]. In fact, it is known that the alteration of bone metabolism in this pathology involves both resorption and bone neoformation (ankylosis, periostitis, and syndesmophytes).

The importance of the role of VD can also be observed in SSc, a connective tissue disease characterized by fibrosis that may involve skin and internal organs [79], in which an altered VD metabolism may increase the risk of OP in association with various factors, such as chronic inflammation, early menopause, immobilization, soft tissue calcification depleting calcium stores [80], and hypothyroidism [81,82]. In SSc patients, 1,25(OH)2D has an antifibrotic effect on fibroblasts, inhibits synthesis of the extracellular matrix, and regulates the skin’s immune system [83], and it is demonstrated that patients with 25(OH)D deficiency have a more severe manifestation of disease than those with 25(OH)D insufficiency [84]. In these patients, common VD supplementation could not correct the deficiency, and a higher dose is required because of the reduced absorption capacity of the thickening intestine [85,86]. However, patients with VD supplementation developed less frequent gastrointestinal ulcers compared with those without treatment [83], which suggests that the supplementation of VD has a therapeutic effect in SSc treatment.

In primary Sjögren’s syndrome (pSS), a chronic autoimmune disease characterized by progressive lymphocyte infiltration of the exocrine glands [87], 25(OH)D and 1,25(OH)2D regulate ocular homeostasis and improve corneal barrier function [88,89]. Indeed, their deficiency increases dry eye disease (DED), which is a manifestation of pSS. VD supplementation improves tear quality, reducing tear osmolarity [90] and the related damage to the ocular surface [91]. Patients with pSS are more prone to OP and fragility fractures, and associations have been observed with aging; female gender [92]; immobilization [93,94]; use of corticosteroids [95]; 25(OH)D insufficiency [96]; renal tubular acidosis [97,98]; and coexistence of other autoimmune diseases, such as primary biliary cholangitis and celiac disease [99]. The alteration of VD metabolism has been documented in pSS patients [100] also due to tubular dysfunction [101], resulting in overt or latent renal tubular acidosis (RTA), and the development of pSS has been associated with some VDR polymorphisms [102]. Furthermore, the association of pSS and VD deficiency increases the probability of osteomalacia and even multiple bone fractures [103,104].

In chronic inflammation conditions of the gastrointestinal tract, including Crohn’s disease (CD), ulcerous colitis (UC), and celiac disease [105], 1,25(OH)2D may also reduce inflammation and maintain gut microbiota, inhibiting the production of inflammatory cytokines in the gastrointestinal tract, the activation of Th1 and Th17 [106], and IL-6 production by epithelial cells [107]. 25(OH)D deficiency is more frequent in these patients than in the general population [108] due to malabsorption, and it may contribute to the development of osteopenia and OP in both UC and CD patients [109]. Therefore, VD supplementation is considered to be an effective and safe therapy in patients with bowel diseases with careful follow-up of 25(OH)D serum levels.

## 3. Allergy, Vitamin D, and OP

**Figure 2:**
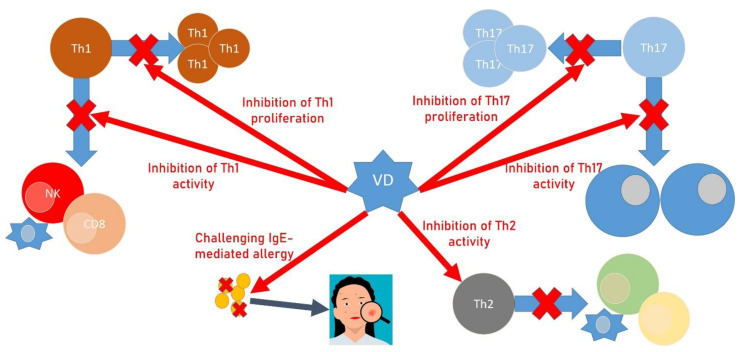
Vitamin D, bone alterations, and allergy. VD acts by skewing T lymphocytes to Th2 polarization and inhibits Th1 and Th17 lymphocyte activity and proliferation.

There is a close connection between allergy and bone metabolism, and the possible negative actions of commonly used therapies are not the only aspects in this relationship. The inflammatory process, its mechanisms, and the cells and molecules involved, as well as VD metabolism, are related to allergic asthma and osteoporosis. A key role in the most common allergic conditions is played by histamine, which is able to stimulate osteoclasts and their precursors in an autocrine and paracrine way [110,111] but also indirectly by enhancing osteoblast expression of RANKL. These pathways are evident, especially in patients affected by systemic mastocytosis (SM), in which diffuse osteopenia and OP with fragility or pathologic fractures are common [112]. Antihistamines seem to act as negative regulators of mesenchymal stem cell differentiation, preventing histamine excess and reducing nontraumatic fractures in user patients [113], and montelukast (CysLTR1 antagonist), clinically used for the treatment of asthma, potently prevents RANKL-induced osteoclast formation and bone loss in vivo [114]. Interestingly, in SM patients, the evidence of high serum levels of sclerostin, a regulator of late osteoblast/pre-osteocyte differentiation associated with osteopenia [115], and 25(OH)D blood values were negatively correlated, and this might suggest that 25(OH)D influences sclerostin levels [115]. There is also a positive association between skeletal health and dairy food intake; young adults with IgE-mediated cow’s milk allergy (IgE-CMA) show a lower bone mineral density (BMD) than that of gender- and age-matched controls. The best source of bioavailable calcium is milk, which also provides proteins important to bone health, such as milk-derived ribonuclease, which promotes angiogenesis within bone tissue, and lactoferrin, which is capable of stimulating osteoblast differentiation and reducing bone resorption. Abnormal BMD scores are found in IgE-CMA patients, but it has to be considered that the IgE-CMA population frequently experiences other manifestations of the atopic march, such as asthma. It is also known that infants with cow milk allergy have lower levels of 25(OH)D [115]. Moreover, 25(OH)D represents an important link between allergy and bone, considering the fact that it plays a leading role in bone metabolism while demonstrating anti-inflammatory activity. VD acts by skewing T lymphocytes to Th2 polarization and inhibits Th1 and Th17 lymphocyte activity and proliferation (Figure 2). It was reported that the association of VD deficiency and asthma, and more specifically with increased airway inflammation, decreased lung function and increased exacerbations and poor prognosis [17,116]. Osteoporosis is also recognized as a long-term sequela of atopic dermatitis (AD). Despite the fact that AD is associated with type 2 inflammation that may result in protection rather than an increased risk of fracture, AD may influence the achievement of a normal peak bone mass due to dietary restrictions with suboptimal calcium and VD intake at a critical period of bone mineralization, mostly when the disease arises early in childhood and is associated with food allergies. In severe AD, it may be difficult to mobilize, and the reduced physical activity contributes to osteopenia [117]. In the literature, 25(OH)D supplementation has been described as beneficial for patients affected by chronic urticaria (CU), although there remains a lack of sufficient evidence [118]. CU is a skin disorder characterized by recurrent wheals and/or angioedema accompanied by itching and a duration of more than 6 weeks [119,120]. One study found that in vitro IgE production is decreased after the assumption that VD [121] in CU could play a role through immunomodulation of IgE-mediated pathways.

## 4. Endocrinological Diseases, Vitamin D and OP

**Figure 3:**
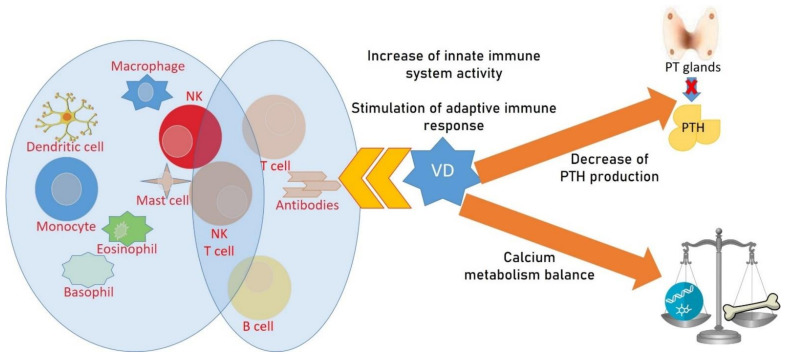
Vitamin D, bone modifications, and endocrinological diseases. VD can increase the activity of the innate immune system and stimulate the adaptive immune response, promoting immune tolerance and reducing the development of autoimmune disease.

Thyroid hormones are essential factors in the regulation of skeletal development and metabolism of the bone. A thyroid pathology can have effects on bone structures, leading to osteoporosis and fragility fractures. While there is a consensus on the adverse effects on bone mass density due to hyperthyroidism, there are not sufficient data regarding hypothyroidism and subclinical conditions. In the state of hyperthyroidism, bone turnover is shorter than in normal conditions [122], with the discrepancy between bone formation and resorption resulting in a net loss of bone. In adults, hypothyroidism is known to reduce bone turnover by decreasing the activity of osteoclasts in bone resorption, but the risk of fracture is not decreased because of the major stiffness of the bone [123]. Furthermore, overtreatment with levothyroxine is a negative factor affecting bone metabolism because it is associated with a lower BMD [124]. Regarding subclinical hypothyroidism, there is no evidence regarding the association with osteoporosis or fragility fractures; rather, subclinical hyperthyroidism treated with radioiodine may increase bone health. The synthesis and secretion of thyroxine (T4) and 3,5,3′-triiodo-l-thyronine (T3) are regulated by the thyroid-stimulating hormone (TSH) axis, and this is negatively regulated by thyroid hormones and cytokines. T3 binds thyroid hormone receptor α (TRα), mainly expressed in the skeleton both on osteoblasts and osteoclasts, as well as hormone receptor β (TRβ), mainly expressed in the pituitary and hypothalamus and involved in the negative feedback control of the hypothalamic–pituitary–thyroid axis. The pituitary gland acts directly on bone cells, such as T3, through the action of TSH on the TSH receptor (TSHR) of osteoblasts and osteoclasts [125,126]. Several studies have demonstrated a relationship between autoimmune thyroid diseases (AITD), Grave’s disease (GD), and Hashimoto’s thyroiditis (HT) and 25(OH)D levels [127,128,129]. VD can increase the activity of the innate immune system and stimulate the adaptive immune response, promoting immune tolerance and reducing the development of autoimmune disease [130,131] (Figure 3). In recent years, several studies have investigated the circulating levels of 25(OH)D in patients affected by AITD, and whether VD deficiency has a role in the pathogenesis of AITD or is only a consequence remains a topic of debate. Further studies may be carried out that determine whether the supplementation of VD modulates the severity of AITD and if VD has a role in non-autoimmune thyroid diseases.

VD deficiency and insufficiency are also common in primary hyperparathyroidism (PHPT). PHPT is an endocrinological disorder characterized by hypercalcemia and elevated serum parathyroid hormone (PTH), often with asymptomatic presentation. RANKL expression is increased in PHPT and is associated with prevalent cortical bone loss and a decrease in BMD in the distal forearm and the hip, which leads to a major risk of fractures [132,133,134,135]. Parathyroidectomy, bisphosphonates, and denosumab may normalize serum calcium levels and increase BMD in patients with PHPT, preventing fracture events [136,137,138]. Data regarding the skeletal effects of VD in PHPT are not conclusive, but VD status did not appear to significantly impact clinical presentation or BMD. Hypovitaminosis D causes secondary hyperparathyroidism. In all patients with osteopenia and OP, frequent dosing of 25(OH)D blood levels is recommended in order to identify secondary hyperparathyroidism, which may require supplementation of 25(OH)D. This strategy allows one to decrease PTH blood values, facilitate optimal calcium absorption, and normalize the calcium bone metabolism [139,140,141].

A low-calcium diet, 25(OH)D insufficiency, physical inactivity, smoking, and genetic predisposition are factors that influence the risk of osteoporosis in type II diabetes (T2DM) patients, associated with an increased risk of fractures resulting from falls. OP is currently one of the major comorbidities of T2DM; in fact, although these patients have a normal BMD, they have a high risk of fracture [142,143,144] that increases with the duration of the disease and poor glycemic control. In particular, age, hyperglycemia, and the accumulation of end-products of advanced glycation (AGE) are able to alter bone quality by acting on the microarchitecture and altering the properties of the matrix [145,146]. Indeed, diabetics have low osteocalcin and high sclerostin levels, especially if sedentary, suggesting reduced bone quality or low bone remodeling in these patients. An association is established between inflammatory biomarkers and the occurrence of T2DM [147]. Particularly, evidence exists that IL-1β is involved in pancreatic β-cell damage and that TNF-α appears to be a key molecule in peripheral insulin resistance [148]. T2DM affects bone health in advanced stages of the disease in many ways that include chronic inflammation resulting in a negative effect on bone architecture and BMD [149]. Often, T2DM and OP coexist, and the drugs used can affect the respective conditions. On the one hand, a healthy lifestyle characterized by a balanced diet and exercise is important for the prevention and treatment of both conditions, while, on the other hand, some treatments, such as metformin, sulfonylureas, dipeptidyl peptidase-4 inhibitors (DPP-4i), and glucagon-like peptide-1 (GLP-1) receptor agonist (GLP-1RA), should be used for the treatment of T2DM concomitant with OP [150].

VD is also implicated in OP secondary to hypercortisolism, a syndrome that determines high cortisol blood levels classified as adrenocorticotropic hormone (ACTH)-dependent or ACTH-independent (i.e., adrenal tumor). The excess endogenous cortisol is the main cause of secondary osteoporosis [151,152,153,154], presenting with pathological fractures that majorly involve the vertebral spine [155]. The mechanism by which excess glucocorticoid leads to the development of secondary osteoporosis is multifactorial and deteriorates both bone quantity and quality, generating a high fracture risk and reducing BMD [156,157]. The decrease in osteoblast number and function seems to play a central role in bone loss, but increased apoptosis of osteocytes, altered autophagy, and changes in RANKL/osteoprotegerin (OPG) and Wnts/sclerostin expression are also involved. Moreover, glucocorticoids reduce intestinal calcium absorption, increase renal excretion [158], and suppress the GH/IGF1 axis and its anabolic effect [159], as well as have adverse effects on muscle strength through increased proteolysis and atrophy of muscle fibers, which are well characterized in Cushing’s disease (CD) and represent risk factors for falls [160,161,162].

Moreover, in CD, the excess glucocorticoids lead to central obesity through increased lipogenesis and adipogenesis. It is well recognized that adipose tissue metabolism is linked to the modulation of bone remodeling [163]. The accumulation of fat in adipose tissue and the overflow of lipids into other tissues create an inflammatory environment that is the basis for severe disorders; in fact, in OP [164], there is a negative relationship between BMD and the rate of visceral adipose tissue/subcutaneous adipose tissue [165]. Patients with hypercortisolism showed higher PTH and phosphates alkaline levels and lower 25(OH)D and osteocalcin values, while serum calcium levels are normal if corrected by albumin concentration, typically lower than those of healthy people. Secondary hyperparathyroidism is found in 25% of patients with hypercortisolism [166]. Higher PTH concentrations in patients with hypercortisolism suggest active bone resorption and secondary hyperparathyroidism [167]. In fact, urinary calcium excretion is high in patients with hypercortisolism, and hypercalciuria might decrease the serum calcium [168], causing parathyroid glands to have increased PTH secretion and, subsequently, stimulating bone resorption. Altogether, the low circulatory levels of 25(OH)D and osteocalcin, involved in bone mineralization under VD modulation, are associated with low lumbar spine BMD, suggesting a deeply negative effect of hypercortisolism on bone mass and quality.

## 5. Osteoporosis, Vitamin D and Monoclonal Gammopathies

Monoclonal gammopathy of uncertain significance (MGUS) is the most common monoclonal gammopathy [169]. MGUS may be classed into non-IgM MGUS and IgM MGUS according to the specific paraprotein produced. Non-IgM MGUS originates from differentiated plasma cells and may evolve in multiple myeloma (MM), while IgM MGUS may progress in lymphoid malignancies, generally Waldenström’s macroglobulinemia, or other different non-Hodgkin lymphomas [169,170]. Rarely, MGUS can be recognized as light chain only, with the exclusive production of gamma or lambda light chains of immunoglobulin [171].

The frequency of MGUS is 3.2% in normal subjects older than 50 years and changes to 7–9% at the age of 85, but only one third of cases are diagnosed [172]. MGUS differs from MM in the presence of serum monoclonal protein <3 g/dL and clonal BM plasma cells <10%, and the lack of organ involvement, such as hypercalcemia, renal failure, anemia, or bone lesions, can be assigned to MGUS [173]. Although the definition of MGUS includes the absence of the lytic bone lesions typical of MM, numerous changes in bone metabolism have been found in these patients. Drake suggested substituting the terminology “monoclonal gammopathy of undetermined significance” with “monoclonal gammopathy of skeletal significance” to indicate the increased characteristic skeletal alterations more precisely in this situation [174].

In fact, in MGUS subjects, the occurrence rate of osteoporosis and fracture is 14%, and the possibility of fracture is twice that in the normal population, principally involving the axial skeleton [175,176,177,178]. Additional confirmation of an increased chance of fractures was sustained by a comorbidity-adjusted Danish population cohort study [179] and by a Swedish registry study that verified a greater possibility for fractures of the axial skeleton in MGUS subjects (2.37 for vertebral/pelvis fractures) [180]. These findings have been corroborated by a meta-analysis of 60,000 subjects, which stated that subjects with MGUS are at greater risk of experiencing vertebral fractures than are healthy controls (RR of 2.50) [178].

Among subjects referred to an osteoporosis hospital, MGUS was identified in 3.6% of patients affected by osteoporosis and only in 2% of the subjects with regular BMD [181]. Subjects with MGUS presented with a more porous cortical and decreased resistance than those of normal subjects [182,183].

Investigations have been conducted in an attempt to ascertain signs within MGUS patients that can suggest greater bone alteration, and older age seems to be more associated with an increased possibility of fractures than is sex [184], while the serum levels of the monoclonal paraprotein are not related with fracture possibility. Instead, the class of the immunoglobulin might be relevant, and the IgA paraprotein subtype has been reported to influence the risk of fractures, although there are contradictory findings if fracture hazard correlates with either kappa or lambda light chain excess [184]. In MGUS subjects, bone modifications seem to be caused by an increased concentration of osteoclast-stimulating elements, such as chemokine ligand 3/macrophage inflammatory protein 1-alpha, and an increase in Dickkopf-related protein 1, an osteoblast-suppressive element, whose gene expression is greater in MGUS plasma cells than in healthy controls [185]. Moreover, in MGUS subjects with fractures, the median RANKL/OPG ratio is considerably increased with respect to median values in MGUS subjects without fractures [177]. However, various other mechanisms have been proposed to explain the onset of osteoporosis and bone fractures in subjects with MGUS, including an alteration in VD.

A potential association between the extent of VD deficiency and the type of gammopathy has also been suggested. In a report, subjects with MGUS and VD deficiency presented an increased incidence of fractures in those with kappa light chains [186]. An even closer correlation was found between VD deficiency and the more severe form of monoclonal gammopathy, MM. Patients with MM have a high presence of bone alterations, comprising osteopenia, osteolytic lesions, and fractures, which can considerably increase the chance of mortality in subjects with MM [187].

**Figure 4:**
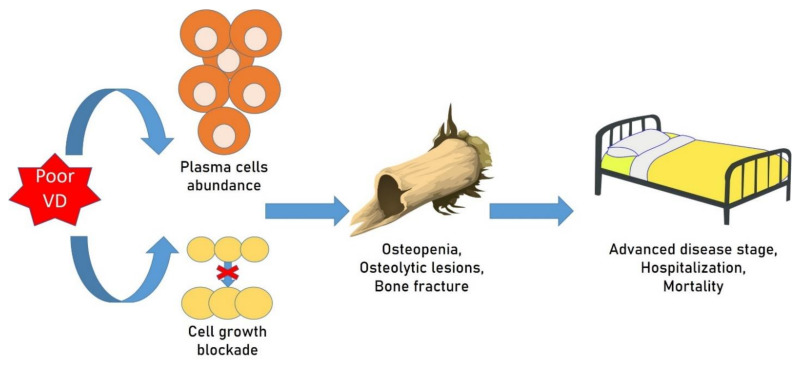
Osteoporosis, vitamin D, and monoclonal gammopathies. VD deficiency influences the onset of an alteration of bone metabolism with secondary osteoporosis or the appearance of bone fractures and lytic lesions. The reduction in VD levels causes the disappearance of the plasma cell proliferation block normally induced by VD.

The negative effect of VD deficiency has been established in MM, with a direct association between reduced plasma VD concentrations and an advanced disease stage in a large group of subjects [188,189]. Similarly, in 2015, German researchers established that more plasma cells in BM are associated with VD concentrations <10 ng/mL [190]. In MM subjects, most of whom presented VD deficiency [191], this association was also confirmed in a long follow-up, where the possibility of MBD onset, osteoporosis, and all-cause mortality seemed small in subjects with adequate VD concentrations (>75 nmol/L) [192]. Finally, in these subjects, VD analogues have been reported to cause cell cycle arrest and programmed cell death [193] (Figure 4).

It is well known that MM cells present VRDs, which are stimulated by nanomolar levels of VD, provoking reduced cell growth [41]. Moreover, in MM cell lines, increased programmed cell death occurs due to the VD analogue EB1089 in the presence of IL-6 and dexamethasone or after administration of VD and other drugs [194,195].

The existence of VD deficiency not only appears to influence the onset of an alteration of bone metabolism with secondary osteoporosis or the appearance of bone fractures and lytic lesions, but it could even be important in provoking the transitions from a benign form of gammopathy, such as MGUS, to neoplastic forms of gammopathies, such as smoldering myeloma (SMM) and MM. In fact, the possibility of progression is increased in subjects with VD deficiency [196].

A study examined the serum VD concentrations of MGUS and SMM patients with the indicators of bone metabolisms, RANKL and OPG, serum protein electrophoresis, and free light chains. Employing these parameters, the authors classified MGUS subjects into two risk progression groups (low or intermediate—risk 1; intermediate or high—risk). Moreover, they demonstrated that oral calciferol administration is followed by a decrease in bone disease progression [197].

However, in spite of the well-known relevance of calcium and VD dispensation in subjects with MGUS, their valuation is still not standardized and has not been proposed to date. Given the findings reported above, these evaluations should become part of the flow chart of data indispensable for the evaluation of MGUS and MM patients, and a reduction should be corrected with oral compounds to preserve calcium homeostasis and bone health. An administration of oral alendronate (70 mg/week) and 1000 mg/daily calcium plus 880 IU/day of VD displayed a decrease in lumbar fractures at 18 months in MGUS with respect to untreated subjects [198,199]. Such an approach could lead to better outcomes for patients with monoclonal gammopathy.

## 6. Bone Marrow Transplantation

Notable bone mineral density loss (BMDL) has been reported in subjects experiencing autologous as well as allogeneic stem cell transplantation (SCT). The genesis of post-bone marrow transplantation (BMT) osteoporosis is a composite, and it may be due to multiple conditions comprising sex, aging, total corticosteroid dosage, immunosuppressive treatments, renal failure, hypogonadism, secondary hyperparathyroidism due to low serum calcium, malabsorption, low body mass index (BMI), and reduced mobility [200,201,202,203,204,205,206]. Previous chemotherapy is also a risk factor as in subjects evaluated after chemotherapy, but before BMT, osteopenia was demonstrated in 24% and osteoporosis in 4% [201]. This action is likely correlated with the effects of treatment on bone marrow stromal cells [207,208]. After BMT bone resorption increases, bone production decreases, causing early, fast bone loss. In addition to osteoporosis, osteomalacia and avascular necrosis may also occur.

Loss in BMD is not a nonthreatening condition. Pundole et al. have reported that the BMD loss in autologous and allogeneic SCT eventually provoked fractures in 8% of transplant subjects at a frequency that was about eightfold increased with respect to that of normal controls [209]. The amount of BMDL is exceptionally fast in the first 4–6 months of transplant, decelerates between 6 and 12 months, and returns to normal afterwards. Improvement starts in the lumbar spine and then in the femoral neck, where BMD nadir is at 24 months [210,211].

Several studies have also displayed the great incidence of VD deficiency in transplant patients, and this is of high relevance, as it constitutes a strong and correctable risk factor [212,213]. Moreover, VD reduction might represent a marker of altered bone health in long-term survivors after allogenic SCT. During follow-up, Baumgartner et al. found that VD deficiency is significantly associated with fractures (hazard ratio (HR) 1.25, 95%CI 1.11–1.41, *p* < 0.001) [214]. In a setting of patients undergoing autologous HCT for MM, 58% of the subjects at 12 months after transplant were stated to be deficient in VD. Analogously, 31% of pediatric recipients were deficient by day 100 post-transplant [215,216,217]. Older age was correlated with an increased risk of maintenance of VD deficiency after HCT [218].

Numerous mechanisms of VD deficiency in HCT have been suggested. VD production from UV light considerably decreases as subjects remain indoors for protracted times for the duration of transplant hospitalization and in the follow-up period, when reduced sun exposure is generally suggested. Moreover, absorption of VD by the intestine can be reduced due to the condition of malabsorption in patients with gastrointestinal graft-versus-host disease (GVHD) and mucositis [219]. A different reason for VD deficiency is constituted by the fact that calcineurin inhibitors and 1,25(OH)2D are both substrates of CYP3A4, and they can interact [220,221]. Finally, hepatic or renal failure caused by conditioning regimen toxicity generally has a role in VD deficiency [222,223].

Recuperation can be deferred up to 10–15 years after transplantation, and several subjects do not recuperate to normal BMD amounts [224]. To evaluate the course of BMDL after SCT in the long term, a study assessed a group of survivors with a follow-up of 12 years. All women took hormone replacement treatment, and standard calcium/VD supplementation was prescribed. BMD considerably increased from 5 to 20 years, but the femoral neck and forearm continued to be susceptible sites. Greater pretransplant BMI, increased BMI post-transplant, and younger age were considerably correlated with increased BMD and provided protection against osteoporosis and osteopenia.

It is now recognized that BMD loss in SCT survivors occurs in two stages, an initial stage of BMD loss (3–5 years) followed by a subsequent stage of BMD improvement. Several elements correlated with therapy and transplant-associated elements, such as VD status, steroids, immunosuppressives, and chronic GVHD, are recognized to influence the initial stage of BMD loss, while age and BMI are more relevant in the stage of BMD recovery [224].

Common suggestions to increase bone health comprise boosting calcium and VD supplementation [225]. Calcium should be administered at a dose of 1000–1200 mg/day of elemental calcium, and when nutritional consumption is inadequate, supplementation with calcium carbonate or citrate is used. Vitamin D3 (VD3) should be administered at a dosage of 1000 IU/day to maintain serum 25(OH)D concentrations of 20–50 ng/mL [226]. However, different dosages have been suggested by various authors [227].

VD and calcium supplementation alone in the initial stage of BMDL cannot prevent BMDL but possibly have a relevant effect on the subsequent stage of BMD recovery [227]. Preventive therapy of osteoporosis should be personalized according to the condition of each patient, and control of BMD for a long time is essential. Hormone substitution treatment is a common therapy in females, while in males, further analysis on gonadal function is necessary [228]. Some authors suggest avoiding bone-protective treatment during the first 3 months after HCT to avoid collateral effects correlated with polypharmacy [229].

The role of osteoporosis and VD and their effects on post-transplant outcomes have been evaluated in several studies focused on their role in immune modulation and GVHD. Campos et al. performed pre-HSCT and 6-month post-HSCT assessments. They evaluated BMD at the lumbar spine and total body. They reported a relevant decrease in BMD 6 months post-HSCT. About 50% of subjects presented a decrease at the LS, and subjects who presented GVHD had the highest diminutions. They also showed that decreases in serum concentrations of 25(OH)D, steroid therapy length, severity of chronic GVHD, and family history of osteoporosis were risk factors correlated with alterations in BMD [230].

Several other studies have confirmed that HCT recipients with VD deficiency are at increased risk of developing acute and chronic GVHD [231,232,233], while some retrospective studies indicated an increased frequency of chronic GVHD in subjects who had pretransplant VD deficiency [8,232].

Preventing and treating VD deficiency may play a role in GVHD prevention and treatment.

## 7. Conclusions

The existence of VDR in most tissues and cells in the body results in a wide range of biological effects of 1α, 25(OH)_2_D in addition to controlling calcium and phosphorus homeostasis. In fact, VD has huge potential, and its possible advantages are under evaluation. However, in spite of the extraordinary advancements made lately, evidence of a correlation between VD and health is scarce, and the results of the studies reported above should be assessed with prudence, as numerous behavioral and lifestyle elements, such as diet, light exposure, age, BMI, season, physical activity, and smoking, can modify serum 25(OH)D amounts. Moreover, the effects of subclinical VD deficiency have to be elucidated, and the possible synergistic effects of VD and micronutrients or antioxidants must be clarified. Finally, another enormous field of study still almost totally unexplored is constituted by the relationship between the VD system and epigenetic mechanisms. In fact, the VD system is controlled by epigenetic mechanisms, but, on the other hand, it is implicated in controlling epigenetic events [226]. Similarly, other studies will be needed to better define the effects of VD in maintaining mitochondrial respiratory functions [234,235] and in the control of normal homeostasis that guarantees well-being.

In summary, it is necessary to recognize that our knowledge of the real relationships between vitamin D and the pathologies we have reported is still very limited, and there is no strong experimental evidence that confirms this association. Despite the above, currently, we do not have a definite cut-off level at which vitamin D could be the cause of several diseases such as those described in this review, and, importantly, we do not have any evidence-based data showing that the prescription of vitamin D (at different dosages) could improve the course of these diseases. Further epidemiological and mechanistic studies are necessary in order to ascertain the real role of hypovitaminosis in causing the reported diseases and to confirm the positive role of vitamin supplementation in prophylaxis and therapy. Particularly, an in-depth study is also necessary when considering the fact that vitamin D is a low-cost drug, one of the most prescribed in Western countries. It is, therefore, necessary to fully clarify the apparently contradictory aspects of the real impact of the deficiency of a widely prescribed substance on the onset of the diseases evaluated in our review.
